# Facile Electrodeposition Preparation of Low-Cost and High-Activity Nickel-Based Hydrogen Evolution Catalysts

**DOI:** 10.3390/nano16100619

**Published:** 2026-05-18

**Authors:** Hai-Chuan Zuo, Guang-Yang Lu, Hai-Bo Yang, Qian Chen, Jian-Ping Zeng, Yong-Gang Sun, Song Chen

**Affiliations:** 1 School of Chemistry and Chemical Engineering, Yancheng Institute of Technology, Yancheng 224051, China; vbgvk060008@163.com (H.-C.Z.); lgy7523@163.com (G.-Y.L.); 18403402662@163.com (H.-B.Y.); cqprcjsyc@163.com (Q.C.); 13770010951@ycit.edu.cn (J.-P.Z.); sunyg86@iccas.ac.cn (Y.-G.S.); 2 Department of Chemistry, Nanjing University of Science and Technology, Nanjing 210094, China

**Keywords:** hydrogen evolution, nickel-based catalysts, multi-field coupled electrodeposition, overpotential, high performance

## Abstract

The hydrogen evolution reaction (HER) of hydrogen production by water electrolysis under alkaline conditions faces enormous challenges, namely, high catalyst overpotential and reliance on noble metal catalysts. As a transition metal, Ni has the advantages of low cost and excellent HER performance. This study aims to develop high-activity and low-cost HER catalysts to replace noble metal catalysts. In this work, a composite structured HER catalyst based on Ni and hybridized with Ni(OH)_2_ and NiO was prepared by multi-field coupled electrodeposition. Under the reversible hydrogen electrode (RHE), low overpotentials of 248 mV and 341 mV were achieved at current densities of 500 mA·cm^−2^ and 1000 mA·cm^−2^, respectively, making it more suitable for industrial high-current-density water electrolysis for hydrogen production. Moreover, the catalyst achieves long-term stable operation at a high current density of 1000 mA⋅cm^−2^ in industrial-grade ALK water electrolysis, with highly stable microstructure and chemical composition before and after the durability test. Theoretical calculations show that compared with the NiM catalyst, Ni@NiM enhances the adsorption capacity for water molecules, further optimizes the ion transport of the catalyst, and the complementarity and synergy in the electronic structure among these multiple components significantly improve the HER activity of the catalyst.

## 1. Introduction

Alkaline water electrolysis for hydrogen production driven by renewable energy is a core supporting technology to realize large-scale green hydrogen production and the “dual carbon” goal, with multiple advantages such as green, low-carbon, sustainability and economy [1]. Compared with the proton exchange membrane water electrolysis system, alkaline water electrolysis can directly use non-noble metal catalysts, which greatly reduces the cost of materials and equipment, and is the mainstream technical route for large-scale hydrogen production in the future [2,3,4,5]. However, the current alkaline water electrolysis technology still faces insurmountable industry pain points: under industrial-grade high current densities above 500 mA·cm^−2^, traditional catalytic materials generally have problems such as sharply increased overpotential, severe bubble coverage, and blocked mass transfer channels, leading to rapid deterioration of reaction kinetics. Especially in the real industrial environment of 30 wt% KOH and 80 °C high temperature, the catalyst is prone to corrosion, dissolution, structural collapse and deactivation of active sites, with extremely poor long-term service stability, which cannot meet the requirements of industrial continuous and stable operation [6,7].

In the alkaline water electrolysis hydrogen production system, noble metal HER catalysts have the advantages of high intrinsic activity, fast reaction kinetics and strong acid–base corrosion resistance, and can achieve efficient hydrogen evolution at low overpotential. Researchers have prepared Ru-doped α-MoC catalysts, which have an overpotential of only 58 mV at 100 mA·cm^−2^, making them a currently excellent material system [8,9,10,11,12,13].

However, the scarcity of noble metal reserves, high prices, and high costs of large-scale application have forced researchers to seek new types of catalysts [14,15,16]. Although transition metal-based catalysts have been extensively studied and can reduce costs to a certain extent, low-cost catalytic systems that can simultaneously achieve high activity, high current density, and stability in high-temperature concentrated alkali are still extremely scarce. Nickel, with the characteristics of abundant reserves, low cost, outstanding intrinsic activity in alkaline media, and excellent corrosion resistance, has become the most promising HER catalytic system to replace noble metals [17,18,19]. Traditional nickel and its alloys do not perform well in electrochemical hydrogen evolution. In recent years, high-performance nickel-based catalysts have been continuously developed. For example, NiFeMoB, composed of multiple metals such as Ni, W, and Mo, has an overpotential of only 218 mV at 500 mA·cm^−2^. The NiCex/NM catalyst with rare earth elements introduced not only has an overpotential of only 169 mV at a high current density of 500 mA·cm^−2^ but also can stably operate in alkaline water electrolysis at a current density of 800 mA·cm^−2^ for more than 2000 h [20,21,22,23,24].

All these prove that nickel-based catalysts have become the most promising research direction to replace noble metal catalysts. While this multi-doping strategy significantly improves catalytic performance, it also brings problems such as increased raw material costs, complicated plating solution composition, and increased difficulty in process control. In addition, nickel-based electrodes prepared by traditional methods such as electrodeposition, coating, and spraying, although capable of achieving excellent performance under laboratory conditions, have their own unavoidable defects: traditional electrodeposition has poor uniformity during large-area preparation and difficult plating solution maintenance; coating is prone to coating cracking, peeling, and poor repeatability; and spraying faces problems such as high porosity, insufficient bonding force, and low material utilization. These limitations make it difficult to transform the excellent performance at the laboratory level into large-scale, long-life industrial applications [25,26,27,28].

More seriously, most of the existing modification strategies involve post-processing and transformation of the formed catalyst structure. Although they can improve HER catalytic activity, it is difficult to simultaneously achieve the synergistic optimization of electrical conductivity, mass transfer capacity, and structural stability during the preparation process, and the methods are cumbersome or require harsh conditions, which is not conducive to large-scale application. As an electrode preparation technology with simple operation, controllable conditions, and easy scaling, electrodeposition has unique advantages in the synthesis of nickel-based HER catalysts [29,30,31]. In recent years, researchers have prepared nickel-based multi-component alloys by electrodeposition, mostly using nickel foam as the substrate. Among them, the CoMo alloy catalyst has an overpotential of only 47 mV at 10 mA·cm^−2^, which improves the catalyst performance on the basis of nickel, but the overall performance is not comparable to that of noble metal catalysts [30,32,33,34].

In addition, the traditional electrodeposition process still faces inherent bottlenecks: limited ion mass transfer under high current density is prone to cause concentration polarization, and the local pH increase caused by hydrogen evolution side reactions is likely to lead to hydroxide inclusions. It is difficult to achieve precise regulation of crystallization behavior and micro-morphology by relying solely on process parameter optimization, thereby restricting the synchronous improvement of the active site density and structural stability of the coating [35,36,37,38]. To break through the above core bottlenecks in the electrodeposition process, it is urgent to effectively optimize and improve the traditional electrodeposition process.

Based on this, this study selects the metal nickel with abundant resources and low cost as the research subject, takes nickel mesh (NiM) as the substrate, and systematically optimizes key parameters such as electrodeposition current density, deposition amount, temperature, pH, flow rate, and solution concentration. A multi-component hybrid structure with high specific surface area, multiple active sites, and high bubble desorption efficiency was directionally constructed, which fundamentally solves the core problems of traditional nickel-based electrodes such as insufficient active sites, low mass transfer efficiency, and poor stability under high current. It achieves an all-round breakthrough in the microstructural optimization, intrinsic activity improvement, and mass transfer capacity acceleration of nickel-based hydrogen evolution electrodes, providing new ideas and technical support for the development of low-cost, high-efficiency, long-life, and scalable alkaline water electrolysis hydrogen evolution electrodes.

## 2. Materials and Methods

### 2.1. Chemical and Materials

Nickel aminosulfonate tetrahydrate (Ni(NH_2_SO_3_)_2_·4H_2_O, Shanghai Zhanyun Chemical Co., Ltd., Shanghai, China), nickel chloride hexahydrate (NiCl_2_·6H_2_O, Shanghai Zhanyun Chemical Co., Ltd., Shanghai, China), potassium hydroxide (KOH, Jiangsu Tongsheng Chemical Reagent Co., Ltd., Jiangsu, China), sodium hydroxide (NaOH, Tianjin Damao Chemical Reagent Factory, Tianjin, China), and sulfuric acid (H_2_SO_4_, 98%, Jiangsu Sopu Chemical Co., Ltd., Jiangsu, China) were the chemical reagents used in this work. All of these reagents were of analytical grade and were used without further purification. A 40-mesh nickel wire mesh (denoted as NiM hereafter, Hebei Kangwei Wire Mesh Co., Ltd., Hebei, China) was used as the substrate. Deionized water was used throughout the experiments.

### 2.2. Materials Fabrication

#### 2.2.1. Pretreatment of Materials

The nickel anode plate was polished with 100-mesh sandpaper until a silvery-white metallic luster appeared, rinsed with deionized water, and dried with a hot air gun. The nickel anode plate and NiM were ultrasonically cleaned in 3 M NaOH at 50 °C for 15 min, rinsed with deionized water, then etched in 1 M H_2_SO_4_ at 50 °C for 10 min, rinsed with deionized water, and dried with a hot air gun. The nickel anode plate was further activated in 10% H_2_SO_4_ for 20 min, rinsed with absolute ethanol and deionized water, and dried with a hot air gun. The NiM had a wire diameter of 150 μm and a mass of 0.08 g·cm^−2^ prior to deposition.

#### 2.2.2. Preparation of Ni@NiM

The treated nickel anode plate and NiM served as the anode and cathode, respectively, in the electrodeposition system. Constant current electrodeposition was performed using a DC power supply, with an electrode distance of 1.5 cm.

### 2.3. Electrochemical Measurement

All tests were performed at room temperature and atmospheric pressure in 1 M KOH (pH = 14) electrolyte using a DH7200A electrochemical workstation with a three-electrode configuration. A platinum sheet and a Hg/HgO electrode (0.098 V vs. RHE) were used as the counter and reference electrodes, respectively. The prepared Ni@NiM was cut to 1 cm^2^ as the working electrode.

The electrode was activated by cyclic voltammetry for 50 cycles between −0.8 and −1.6 V at 100 mV·s^−1^. Linear sweep voltammetry was then performed at 5 mV·s^−1^ with 95% IR compensation. The overpotentials at 500 mA·cm^−2^ and 1000 mA·cm^−2^ (after impedance calibration) were used to evaluate the HER catalytic activity.

## 3. Results

### 3.1. Exploration of Electrodeposition Factors

**Figure 1:**
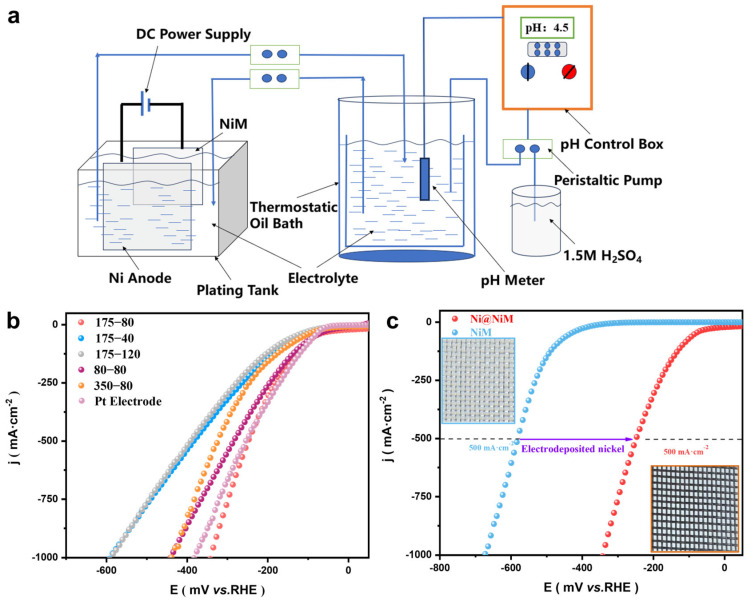
(**a**) Schematic diagram of the electrodeposition system; (**b**) LSV curves of Ni@NiM under different process conditions (expressed by x-y, where x is the concentration of Ni(SO_3_NH_2_)_2_·4H_2_O, g·L^−1^; y is the current density, mA·cm^−2^); and (**c**) LSV curves of Ni@NiM and NiM.

The nickel concentration in the electrodeposition solution usually determines the morphology and growth quality of the catalyst. Different from most researchers, this study used 175 g·L^−1^ Ni(SO_3_NH_2_)_2_·4H_2_O (nickel aminosulfonate) and 10 g·L^−1^ NiCl_2_·6H_2_O (nickel chloride hexahydrate) as the main components of the electrodeposition solution to balance the internal stress control and conductivity optimization of the coating. The electrodeposition system design is shown in Figure 1a. Using the pretreated NiM (nickel mesh) as the substrate, the electrodeposition temperature was set to 50 °C, the deposition amount was 0.1 g·cm^−2^, the pH value was 4.5~5, and the flow rate was 0.75 L·min^−1^; the concentration of NiCl_2_·6H_2_O was kept constant during the fabrication of all electrodeposited samples. The current density determines the intensity of the deposition driving force. This study abandoned the low current density used in traditional nickel electrodeposition and controlled the current density at 80 mA·cm^−2^ to obtain high-density active sites.

The HER performance of Ni@NiM was evaluated in a three-electrode system composed of 1 M KOH at 25 °C, as shown in Figure 1b,c. The overpotentials of Ni@NiM at current densities of 500 mA·cm^−2^ and 1000 mA·cm^−2^ were 248 mV and 341 mV, respectively, indicating good electrochemical hydrogen evolution performance. In terms of electrochemical performance, Ni@NiM exhibited hydrogen evolution activity comparable to or even partially exceeding that of noble metal catalysts. Its low overpotential performance at high current densities effectively broke through the technical bottleneck of insufficient intrinsic activity of non-noble metal catalysts. Moreover, it completely eliminated the introduction of noble metal components such as platinum and ruthenium, significantly reducing the raw material cost. In terms of the preparation process, compared with the nickel-based composites widely reported in the literature, Ni@NiM was prepared in one step by a simple electrodeposition method, without a high-temperature and high-pressure environment, complex precursor synthesis or tedious post-treatment procedures, which greatly simplified the production process. Compared with traditional nickel-based alloys, Ni@NiM did not introduce a second metal element and exhibited extremely high HER activity through the optimized nanoneedle array structure and surface chemical state.

**Figure 2:**
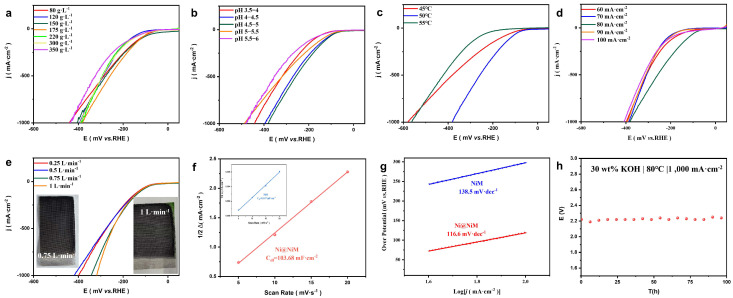
(**a**) LSV curves of Ni@NiM prepared with different concentrations of Ni(SO_3_NH_2_)_2_·4H_2_O; (**b**) LSV curves of Ni@NiM prepared at different pH values; (**c**) LSV curves of Ni@NiM prepared at different temperatures; (**d**) LSV curves of Ni@NiM prepared at different current densities; (**e**) LSV curves of Ni@NiM prepared at different flow rates and their corresponding appearance diagrams; (**f**) C_dl_ values of Ni@NiM and NiM; (**g**) Tafel curves of Ni@NiM and NiM; and (**h**) voltage variation curve of Ni@NiM in ALK water electrolysis for 100 h.

To further explore the effects of deposition solution concentration, temperature, deposition amount, pH, and flow rate on Ni@NiM, a series of single-factor experiments was designed (Figure 2a–d) with 175 g·L^−1^ Ni(SO_3_NH_2_)_2_·4H_2_O, 10 g·L^−1^ NiCl_2_·6H_2_O, pH 4.5~5, temperature 50 °C, current density 80 mA·cm^−2^, and deposition amount 0.1 g·cm^−2^ as the standard, and the rationality of each parameter design was verified. We observed that as the nickel aminosulfonate concentration increased from 80 to 175 g·L^−1^, the surface morphology evolved from rough rock-like structure into fine, dense nanoneedles with compact stacking, which greatly increased the active sites for HER. By contrast, when the concentration further rose to 350 g·L^−1^, the nanoneedles started to agglomerate. In particular, at 300 g·L^−1^, the needle-like structure gradually faded and was replaced by coarse block-like morphology, leading to reduced active sites (Figure S1). Moreover, the sample prepared at room temperature (25 °C) presents a turquoise surface and poor mechanical strength, making it unsuitable for water electrolysis applications (Figure S2). We found that neither excessively high nor low current densities are appropriate for electrodeposition. Under an ultralow current density, the catalyst surface is relatively flat, leading to a substantial decrease in catalytic activity. By contrast, an overly high current density triggers structural collapse and particle fusion on the surface, resulting in severe degradation of the catalytic performance (Figure S3).

On this basis, a flow field was introduced (Figure 2e) to accelerate mass transfer efficiency for obtaining higher-activity Ni@NiM. It can be observed that when the flow rate was 1 L·min^−1^, Ni@NiM exhibited the optimal hydrogen evolution performance in electrochemical tests; however, the electrode showed an obvious greenish discoloration in appearance. This is because the excessively high circulation flow rate led to excessively high local current density at the electrode edges due to the “edge effect”, which intensified the hydrogen evolution side reaction, significantly increased the local pH value, and further generated green Ni(OH)_2_. Although this green coating may increase the electrode surface area to a certain extent or provide different active phases, its long-term stability is questionable, and the coating uniformity is seriously substandard. Therefore, the optimal flow rate is 0.75 L·min^−1^. To verify the experimental repeatability, we conducted repeated experiments, and the results confirm that our synthesis method presents good repeatability, as well as good uniformity of the prepared samples (Figure S4). In addition, Ni@NiM has an extremely high C_dl_ value (103.68 mF·cm^−2^), which provides more active area compared with NiM (0.347 mF·cm^−2^) (Figure 2f).

In the electrochemical measurements, the ECSA is proportional to the C_dl_, and their relationship is shown in Equation (1). The ECSA value of Ni@NiM is 1481 cm^2^, while that of NiM is only 8.7 cm^2^, demonstrating that the catalyst achieves a considerable electrochemical activity after electrodeposition. Furthermore, the charge transfer resistance is inversely proportional to the exchange current density, and the latter follows the quantitative relationship with the Tafel slope as presented in Equation (2). Based on this correlation, we calculated the exchange current densities of Ni@NiM and bare NiM to be 9.54 mA·cm^−2^ and 0.02 mA·cm^−2^ (Figure 2g), respectively. This result clearly demonstrates that Ni@NiM exhibits significantly enhanced charge transfer capability [39]. To verify the stability of Ni@NiM under industrial-grade ALK water electrolysis conditions, we tested the catalyst at a constant current density of 1A·cm^−2^ in 30 wt% KOH at 80 °C for 100 h. The voltage evolution during the test is shown in Figure 2h, and no obvious voltage decay was observed throughout the process, demonstrating the excellent long-term stability of the catalyst.

(1) $$\mathrm{ECSA}={\;C}_{\mathrm{dl}}/C_{s}$$

(2) $$\eta =\mathrm{blg}(j/j_{0})$$

### 3.2. Material Characterization

**Figure 3:**
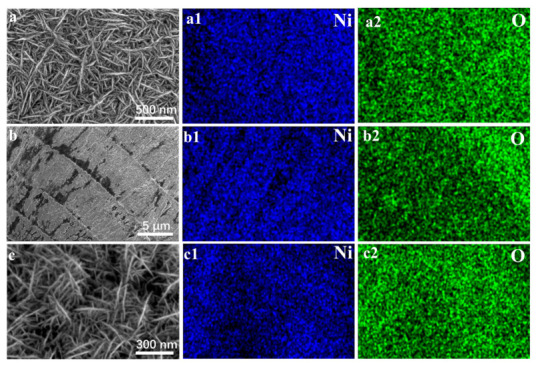
(**a**,**a1**,**a2**) Surface SEM and EDS images of Ni@NiM; (**b**,**b1**,**b2**) cross-sectional SEM and EDS images of Ni@NiM; and (**c**,**c1**,**c2**) surface SEM and EDS images of Ni@NiM after 100 h alkaline water electrolysis.

The unique high current density electrodeposition process adopted has a significant impact on the surface morphology and composition structure of Ni@NiM. SEM characterization results show that Ni@NiM presents a fine needle-like structure without obvious large-scale aggregation, and the overall deposition effect is good. Further observation reveals that the needles on the electrode surface are closely stacked but retain certain gaps. This structural feature not only provides abundant catalytic active sites but also does not cover the catalytic activity of the catalyst under the surface layer, realizing the efficient utilization of active sites. It is this special needle-like stacked structure that creates a favorable steric hindrance environment for multi-component hybridization (Figure 3a). It can be seen from Figure 3(a1,a2) that Ni and O are uniformly distributed on the surface of Ni@NiM. In addition, the cross-section of Ni@NiM presents a layered, stacked rock-like shape (Figure 3b), indicating that Ni@NiM is a high specific surface catalyst stacked by nano-nickel needles. The uniform distribution of Ni and O in the cross-sectional energy spectrum further verifies the high uniformity of the Ni@NiM coating (Figure 3(b1,b2)).

As depicted in Figure 3c, the microstructure of the Ni@NiM catalyst is well preserved after 100 h of continuous alkaline water electrolysis under industrial-relevant conditions. The overall nanoneedle-like framework remains intact without obvious collapse or agglomeration. No significant coarsening is observed in the needle structure compared with the fresh sample. Meanwhile, no severe etching pits or exfoliation induced by continuous bubble release and high-flow electrolyte erosion can be detected on the surface. Furthermore, the elemental distribution on the catalyst surface remains highly uniform after the long-term durability test (Figure 3(c1,c2)). Such outstanding morphological stability firmly validates the robust structural stability of Ni@NiM toward industrial-scale water electrolysis applications.

**Figure 4:**
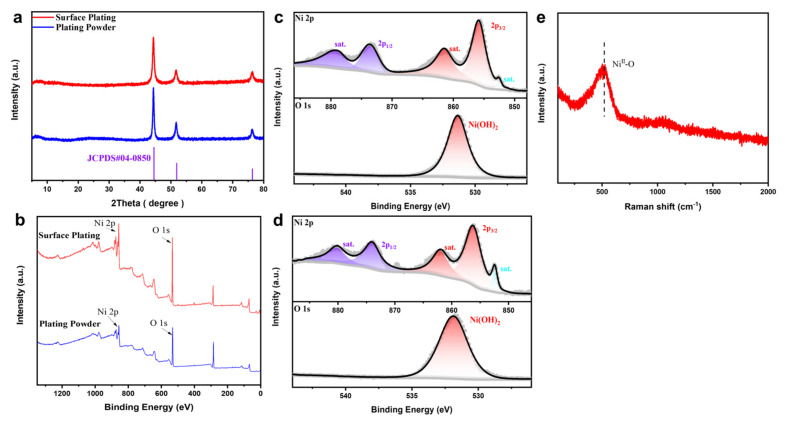
(**a**) XRD patterns of the surface coating and coating powder of Ni@NiM; (**b**) XPS survey spectra of the surface coating and coating powder of Ni@NiM; (**c**) Ni 2p and O 1s orbital spectra of the surface coating of Ni@NiM; (**d**) Ni 2p and O 1s orbital spectra of the coating powder of Ni@NiM; and (**e**) Raman spectrum of Ni@NiM.

Metals are usually accompanied by the formation of oxide films in the air, and the hydrogen evolution reaction is almost unavoidable during the electrodeposition process, so the coating is often accompanied by the formation of hydroxides. The Ni@NiM prepared in this experiment is no exception, so Ni(OH)_2_ must exist in the catalyst. The surface and powder of the Ni@NiM coating were characterized. As shown in Figure 4a, in the XRD pattern, the diffraction peaks at 44.5°, 51.8°, and 76.4° correspond to the (1 1 1), (2 0 0), and (2 2 0) crystal planes of Ni (JCPDS#04-0850) [40,41,42], respectively. No typical diffraction peaks of other compounds were observed, which obviously cannot indicate that there are no other substances such as Ni(OH)_2_ and NiO in Ni@NiM. XPS was used to analyze Ni@NiM, and the test results are shown in Figure 4b–d: in the high-resolution XPS spectrum of Ni 2p, the peaks observed at the binding energies of 856 eV and 873 eV on the surface coating correspond to the characteristic signals of Ni^2+^; the binding energies of 861.6 eV and 879.1 eV correspond to the satellite peaks of Ni^2+^, and a satellite peak corresponding to Ni^0^ was observed at 852.5 eV, indicating that nickel mainly exists in the form of Ni^2+^ in the surface coating. In addition, it can be seen from the high-resolution XPS spectrum of O 1s that Ni(OH)_2_ exists in the surface coating [43]. The coating powder is similar to the surface coating, except that the peaks of Ni^0^ in its Ni 2p spectrum are more abundant [44], which indicates that the occurrence of the hydrogen evolution reaction during the electrodeposition process covers the Ni surface with a layer of Ni(OH)_2_, providing multiple hydrogen evolution sites for the catalyst.

Similarly, there may be NiO in Ni@NiM that is not detected by XRD. Therefore, a Raman test was performed on Ni@NiM, and the test results are shown in Figure 4e, confirming the presence of NiO in the sample [45,46]. This multi-component structure co-constructed by Ni, NiO and Ni(OH)_2_ realizes the regulation of electronic structure and optimization of reaction path through the interfacial charge transfer and lattice matching effect between various components, synergistically improving the overall hydrogen evolution activity of the catalyst. Furthermore, the chemical composition of Ni@NiM remained almost unchanged after long-term ALK water electrolysis (Figures S5 and S6).

**Figure 5:**
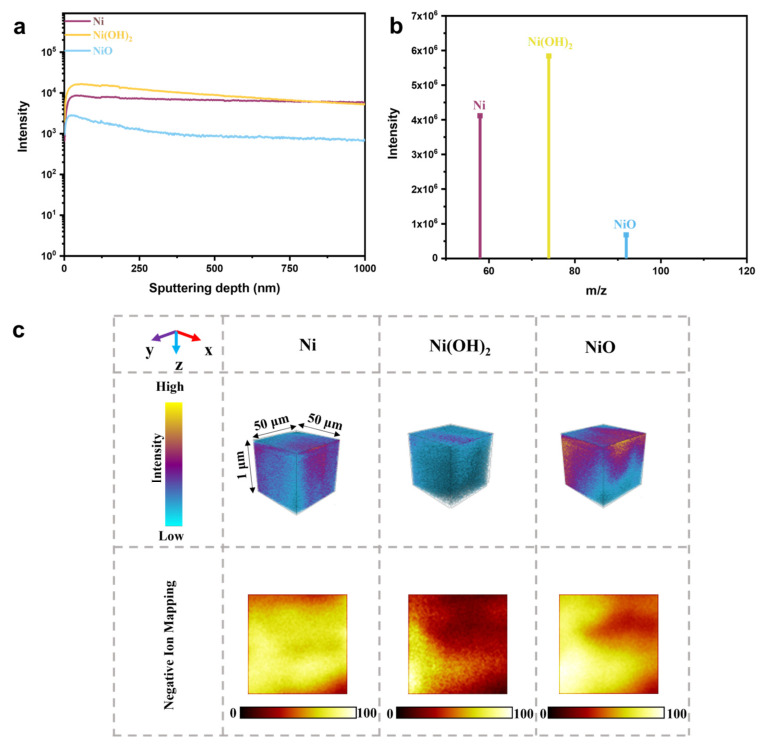
(**a**) Curve of signal change in each component in Ni@NiM with etching time; (**b**) mass-to-charge ratio of each component in Ni@NiM; and (**c**) spatial distribution diagram of each component in Ni@NiM.

To further explore the composition and spatial distribution characteristics of Ni@NiM, a time-of-flight secondary ion mass spectrometry (TOF-SIMS) test was performed on it. The test results show that the characteristic ion signal intensities corresponding to Ni, Ni(OH)_2_, and NiO do not show obvious enhancement or attenuation trends within the entire etching depth range (Figure 5a,b), and it can be intuitively seen that these three components are uniformly distributed along the depth direction in the coating (Figure 5c), indicating that Ni@NiM is a multi-component hybrid catalyst composed of Ni, Ni(OH)_2_, and NiO together. This structural feature not only promotes ion transport and interfacial charge redistribution inside the coating but also provides more abundant active sites and an optimized reaction microenvironment for the hydrogen evolution reaction.

### 3.3. Research on the HER Mechanism

**Figure 6:**
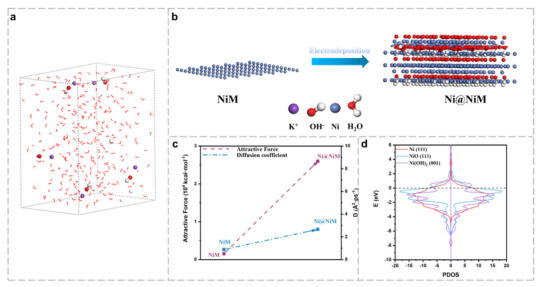
(**a**) 1 M KOH solution model; (**b**) NiM and Ni@NiM electrode models; (**c**) interaction forces between NiM, Ni@NiM and water molecules in 1 M KOH; and (**d**) PDOS diagrams of Ni, Ni(OH)_2_ and NiO.

Based on various characterization results, it is confirmed that Ni@NiM is a multi-component hybrid catalyst composed of Ni, Ni(OH)_2_ and NiO, and exhibits excellent electrochemical performance. To clarify the internal mechanism of the components synergistically enhancing HER activity, the interaction behavior between Ni@NiM and water molecules in a 1 M KOH system was simulated. Comparative analysis shows that the attraction of Ni@NiM to water molecules is greatly enhanced, and the diffusion capacity of water molecules is about three times that of a pure nickel electrode [47,48]. The interfacial synergistic effect formed between different phases accelerates the generation of active hydrogen in the Volmer step (Figure 6a–c).

Density functional theory calculations were carried out, and the results show (Figure 6d) that the d-band center of the 3d orbital of Ni is −0.89 eV, while the d-band centers of Ni in NiO and Ni(OH)_2_ are 0.44 eV and −0.92 eV respectively. Compared with Ni, the d-band center of Ni in NiO shifts upward, which helps to weaken the interaction between HER reaction intermediates and active sites, reduce the adsorption free energy, and thus accelerate the reaction kinetics [49,50]. In this study, the content of Ni is relatively less than that of Ni(OH)_2_, but it still shows very excellent hydrogen evolution activity. This is because during the high current density deposition of Ni@NiM, Ni, Ni(OH)_2_ and NiO form abundant heterogeneous interfaces, resulting in interfacial synergistic effects. It is precisely the complementarity and synergy between the multi-components of Ni, Ni(OH)_2_ and NiO in electronic structure that enable the catalyst to achieve better overall hydrogen evolution reaction kinetics even when the proportion of Ni is relatively reduced.

## 4. Discussion

The catalyst prepared under the conditions of 175 g·L^−1^ Ni(SO_3_NH_2_)_2_·4H_2_O, 10 g·L^−1^ NiCl_2_·6H_2_O, pH 4.5~5, temperature 50 °C, current density 80 mA·cm^−2^, deposition amount 0.1 g·cm^−2^, and flow rate 0.75L·min^−1^ exhibits excellent HER performance in alkaline medium: the overpotentials at high current densities of 500 mA·cm^−2^ and 1000 mA·cm^−2^ are only 248 mV and 341 mV, respectively. Moreover, the catalyst achieves long-term stable operation at a high current density of 1000 mA⋅cm^−2^ in industrial-grade ALK water electrolysis, with highly stable microstructure and chemical composition before and after the durability test. Characterization results further reveal that the composite catalyst Ni@NiM, composed of Ni, Ni(OH)_2_ and NiO, forms abundant heterogeneous interfaces. Molecular dynamics simulation results show that in alkaline solution, the adsorption capacity of Ni@NiM for water molecules is significantly stronger than that of Ni, and the diffusion coefficient of water molecules on its surface is about three times that of Ni, which is conducive to accelerating the reaction kinetics of the Volmer step under alkaline conditions. DFT calculations show that the complementarity and synergy in electronic structure between the multi-components of Ni, Ni(OH)_2_ and NiO significantly improve the hydrogen evolution activity of Ni@NiM.

## 5. Conclusions

This study developed a special multi-field coupled electrodeposition technology under high current density and successfully synthesized a low-cost and high-activity Ni@NiM catalyst multi-hybridized by Ni, Ni(OH)_2_ and NiO. It exhibits excellent HER performance in alkaline medium, which is significantly better than that of a pure nickel electrode under the same test conditions, reflecting its application potential in the industrial electrolytic water hydrogen production scenario with high current density. This study not only systematically reveals the electronic structure and interfacial interaction mechanism of Ni@NiM multi-components synergistically enhancing HER reaction at the micro level, but also provides new theoretical guidance and preparation strategy for the development of efficient, stable and low-cost nickel-based non-noble metal HER catalysts.

## Data Availability

Data are contained within the article.

## Supplementary Materials

The following supporting information can be downloaded at: https://www.mdpi.com/article/10.3390/nano16100619/s1.

## References

[1] Wang Z. (2025). Analysis of Progress and Optimization Pathways for Hydrogen Production Technologies under Dual Carbon Goals. Appl. Comput. Eng..

[2] Shehzad B., Latif S., Wahab A., Shafique M.U., Ch A.R. (2026). Progress and challenges in alkaline water electrolysis for sustainable hydrogen production. Discov. Chem..

[3] Chu C.C., Suhainin M.D., Pg Haji Omar Ali D.N.H.A., Lim J.Y., Swee P.S., Raymundo J.Y., Tan R.X.H., Yap M.K., Khoo H.F., Suhaimi H. (2025). A Techno-Economic Assessment of Steam Methane Reforming and Alkaline Water Electrolysis for Hydrogen Production. Hydrogen.

[4] Sebbahi S., Assila A., Alaoui Belghiti A., Laasri S., Kaya S., Hlil E.K., Rachidi S., Hajjaji A. (2024). A comprehensive review of recent advances in alkaline water electrolysis for hydrogen production. Int. J. Hydrogen Energy.

[5] Esfandiari N., Aliofkhazraei M., Colli A.N., Walsh F.C., Cherevko S., Kibler L.A., Elnagar M.M., Lund P.D., Zhang D., Omanovic S. (2024). Metal-based cathodes for hydrogen production by alkaline water electrolysis: Review of materials, degradation mechanism, and durability tests. Prog. Mater. Sci..

[6] Zhao Z., Sun H., Li J., Sun Z., Guo Y. (2026). Key three-material system in alkaline water electrolysis for hydrogen production: Systematic review and future outlook. Fuel.

[7] Tüysüz H. (2024). Alkaline Water Electrolysis for Green Hydrogen Production. Acc. Chem. Res..

[8] Zhao Q., Chen G., Yang J., Shen Q., Cheng Y., Gu H., Zhang J., Guo Z., Zhao Y. (2026). Boosting hydrogen evolution reaction performance via Pt-loaded phytic acid-etched NiCo-MOF electrocatalysts. Appl. Surf. Sci..

[9] Yao Y., Chen X., Shi M., Qiu Y., Wu D., Yan H., Liu L. (2025). Effect of Ni–Ru/CF electrode fabrication and wettability modification on hydrogen evolution reaction and bubble evolution behavior during water electrolysis. Int. J. Hydrogen Energy.

[10] Zhang X., Wang H., Dai R., Zhao P., Wang Y. (2025). MXene and Ru doping co-enhanced the hydrogen evolution reaction performance of cobalt pyridinedicarboxylic coordinated polymer. J. Colloid Interface Sci..

[11] Yao J., Wang J., Wang W., Xu H., Chen D., Li G., Jin Z. (2026). Precision-Engineered Electronic Modulation of Ruthenium Clusters and Single Atoms on Vacancy-Rich α-MoC_1−x_ Enables Efficient Electrocatalytic Water Splitting. Adv. Mater..

[12] Yu M., Ye Q., Wang F., Abdukayum A., Li N., Zhang L., Zuo C., Liu W., Zhao X., Hu G. (2026). Ternary metal NiRuPt partition synergistic relay promotes pH-universal hydrogen evolution. Nano Res..

[13] Jin D., Zhai Y., Huang Y., Wang L., Ye Y., Ge J., Li G. (2026). Multiscale Synergistic Design of High-Performance Electrocatalysts for Alkaline Hydrogen Evolution. Small.

[14] Khan M.A., Zhao H., Zou W., Chen Z., Cao W., Fang J., Xu J., Zhang L., Zhang J. (2018). Recent Progresses in Electrocatalysts for Water Electrolysis. Electrochem. Energy Rev..

[15] Wang F., Xiao L., Jiang Y., Liu X., Zhao X., Kong Q., Abdukayum A., Hu G. (2025). Recent achievements in noble metal-based oxide electrocatalysts for water splitting. Mater. Horiz..

[16] Wang S., Lu A., Zhong C.-J. (2021). Hydrogen production from water electrolysis: Role of catalysts. Nano Converg..

[17] Gong M., Wang D.-Y., Chen C.-C., Hwang B.-J., Dai H. (2016). A mini review on nickel-based electrocatalysts for alkaline hydrogen evolution reaction. Nano Res..

[18] Danilovic N., Subbaraman R., Strmcnik D., Chang K.-C., Paulikas A.P., Stamenkovic V.R., Markovic N.M. (2012). Enhancing the Alkaline Hydrogen Evolution Reaction Activity through the Bifunctionality of Ni(OH)_2_/Metal Catalysts. Angew. Chem. Int. Ed..

[19] Miao D., Li J., Ren J., Chen Z. (2026). Exploring the Potential of Ni-Based Hydrogen Evolution Catalysts in Anion Exchange Membrane Water Electrolyzer. Adv. Mater..

[20] Yang X., Leng L., Zhang G., Deng Z., Cheng J. (2026). Amorphous NiS deposited on crystalline Co_3_O_4_ nanosheets boosting electron transfer for efficient hydrogen evolution. J. Power Sources.

[21] Wang Z., Tan X., Wang C., Li G., Yuan S. (2025). Mo-doped Ni/NiO as a highly efficient and stable electrocatalyst for the hydrogen evolution reaction. Int. J. Hydrogen Energy.

[22] Wang A., Long G., Chen J., An X., Yao T., Li C. (2024). Electrocatalysts with Highly Dispersed Cerium in Nickel Matrix for Hydrogen Evolution in Alkaline Electrolyte. Adv. Energy Mater..

[23] Chen S., Zhang J., Xu C., Tan X., Liang J., Guo Y. (2025). Practical Monolithic W, Mo Dual-Doped NiFeB Catalyst for Overall Water Splitting. ACS Appl. Energy Mater..

[24] Liu X., Zhang W., Wu X., Cho Y.-R. (2025). Porous Fe-Doped Ni_3_P/CoP_3_ Isomerism as High Durable Electrocatalyst for Generation of Hydrogen and Oxygen. Energy Mater. Adv..

[25] Ďurovič M., Hnát J., Bouzek K. (2021). Electrocatalysts for the hydrogen evolution reaction in alkaline and neutral media. A comparative review. J. Power Sources.

[26] Zhai W., Ma Y., Chen D., Ho J.C., Dai Z., Qu Y. (2022). Recent progress on the long-term stability of hydrogen evolution reaction electrocatalysts. InfoMat.

[27] Kang W.-J., Feng Y., Li Z., Yang W.-Q., Cheng C.-Q., Shi Z.-Z., Yin P.-F., Shen G.-R., Yang J., Dong C.-K. (2022). Strain-Activated Copper Catalyst for pH-Universal Hydrogen Evolution Reaction. Adv. Funct. Mater..

[28] Xu S., Zhang P., Li L., Moon M.-W., Chung C.-H., Li H., Lee J.Y., Yoo P.J. (2025). Challenges and Emerging Trends in Hydrogen Energy Industrialization: From Hydrogen Evolution Reaction to Storage, Transportation, and Utilization. Small.

[29] Kim J., Kim H., Han G.H., Hong S., Park J., Bang J., Kim S.Y., Ahn S.H. (2022). Electrodeposition: An efficient method to fabricate self-supported electrodes for electrochemical energy conversion systems. Exploration.

[30] Chae M.J., Suh S.J. (2026). FeCoNiWV-based catalysts via pulse electrodeposition for high-performance hydrogen evolution. Appl. Surf. Sci..

[31] Miao M., Duan H., Luo J., Wang X. (2022). Recent progress and prospect of electrodeposition-type catalysts in carbon dioxide reduction utilizations. Mater. Adv..

[32] Jian X., Zhang W., Yang Y., Li Z., Pan H., Gao Q., Lin H.-J. (2024). Amorphous Cu–W Alloys as Stable and Efficient Electrocatalysts for Hydrogen Evolution. ACS Catal..

[33] Friedman A., Yang K., Ge H., Mukerjee S. (2025). Advanced Deposition Methods for Mixed Metal Alloys and Hydroxides as High-Performance Catalysts for the Hydrogen Evolution Reaction. ACS Catal..

[34] Guo D., Duan D., Gao J., Zhou X., Liu S., Wang Y. (2022). Synthesis of nest-like porous MnCo–P electrocatalyst by electrodeposition on nickel foam for hydrogen evolution reaction. Int. J. Hydrogen Energy.

[35] Kamble C., Jadhav V.V., Mane R.S., Mane R., Jadhav V., Al-Enizi A. (2023). Chapter 11—Electrodeposition of metal oxide nanostructures. Solution Methods for Metal Oxide Nanostructures.

[36] Zielinski A., Bartmanski M. (2020). Electrodeposited Biocoatings, Their Properties and Fabrication Technologies: A Review. Coatings.

[37] Lee S.-Y., Kim S.-J., Lee W.J., Lee Y.-K. (2021). Boosting Activity and Durability of an Electrodeposited Ni(OH)_2_ Catalyst Using Carbon Nanotube-Grafted Substrates for the Alkaline Oxygen Evolution Reaction. ACS Appl. Nano Mater..

[38] Xu X., Jiao X., Kapitanova O.O., Wang J., Volkov V.S., Liu Y., Xiong S. (2022). Diffusion Limited Current Density: A Watershed in Electrodeposition of Lithium Metal Anode. Adv. Energy Mater..

[39] de Chialvo M.R.G., Chialvo A.C. (1996). The polarisation resistance, exchange current density and stoichiometric number for the hydrogen evolution reaction: Theoretical aspects. J. Electroanal. Chem..

[40] Main R.M., Vornholt S.M., Rice C.M., Elliott C., Russell S.E., Kerr P.J., Warren M.R., Morris R.E. (2023). In situ single-crystal synchrotron X-ray diffraction studies of biologically active gases in metal-organic frameworks. Commun. Chem..

[41] Li S., Li M., Ni Y. (2020). Grass-like Ni/Cu nanosheet arrays grown on copper foam as efficient and non-precious catalyst for hydrogen evolution reaction. Appl. Catal. B Environ. Energy.

[42] Saad A., Bai L., Christensen F.M.S., Luo S., Bentien A., Ashokkumar M., Wei Z. (2025). Ultrasound-enhanced alkaline water splitting with fast bubble release and sustained Ni catalysts. Appl. Catal. B Environ. Energy.

[43] Kuang W., Cui Z., Wang C., Chen T., Wang Q., Li S., Yang T., Liu J. (2025). Self-Supported Ni/Ni(OH)_2_ Electrodes for High-Performance Alkaline and AEM Water Electrolysis. Adv. Energy Mater..

[44] Zhang P., Zhao Y., Li Y., Li N., Silva S.R.P., Shao G., Zhang P. (2023). Revealing the Selective Bifunctional Electrocatalytic Sites via In Situ Irradiated X-Ray Photoelectron Spectroscopy for Lithium–Sulfur Battery. Adv. Sci..

[45] Saguì N.A., Ström P., Edvinsson T., Bayrak Pehlivan İ. (2022). Nickel Site Modification by High-Valence Doping: Effect of Tantalum Impurities on the Alkaline Water Electro-Oxidation by NiO Probed by Operando Raman Spectroscopy. ACS Catal..

[46] Steimecke M., Seiffarth G., Schneemann C., Oehler F., Förster S., Bron M. (2020). Higher-Valent Nickel Oxides with Improved Oxygen Evolution Activity and Stability in Alkaline Media Prepared by High-Temperature Treatment of Ni(OH)_2_. ACS Catal..

[47] Wang B., Gao H., Wu H., Wu Y., Ren B., Liu X., Nie Y. (2024). Diffusion coefficients during regenerated cellulose fibers formation using ionic liquids as solvents: Experimental investigation and molecular dynamics simulation. Chem. Eng. J..

[48] Ma L., Salehi H.S., Jing R., Erkens S., Vlugt T.J.H., Moultos O.A., Greenfield M.L., Varveri A. (2023). Water diffusion mechanisms in bitumen studied through molecular dynamics simulations. Constr. Build. Mater..

[49] Tang T., Liu X., Luo X., Xue Z., Pan H.-R., Fu J., Yao Z.-C., Jiang Z., Lyu Z.-H., Zheng L. (2023). Unconventional Bilateral Compressive Strained Ni–Ir Interface Synergistically Accelerates Alkaline Hydrogen Oxidation. J. Am. Chem. Soc..

[50] Zhang C., Liang X., Xu R., Dai C., Wu B., Yu G., Chen B., Wang X., Liu N. (2021). H_2_ In Situ Inducing Strategy on Pt Surface Segregation over Low Pt Doped PtNi_5_ Nanoalloy with Superhigh Alkaline HER Activity. Adv. Funct. Mater..

