# Supplementary material for: Facile Electrodeposition Preparation of Low-Cost and High-Activity Nickel-Based Hydrogen Evolution Catalysts
Source: Nanomaterials (Basel). 2026 May 18;16(10):619. doi: 10.3390/nano16100619 (PMC13209527; doi:10.3390/nano16100619)
Supplement: Supplementary file 1 [file nanomaterials-16-00619-s001.zip › nanomaterials-4283154-supplementary.pdf]

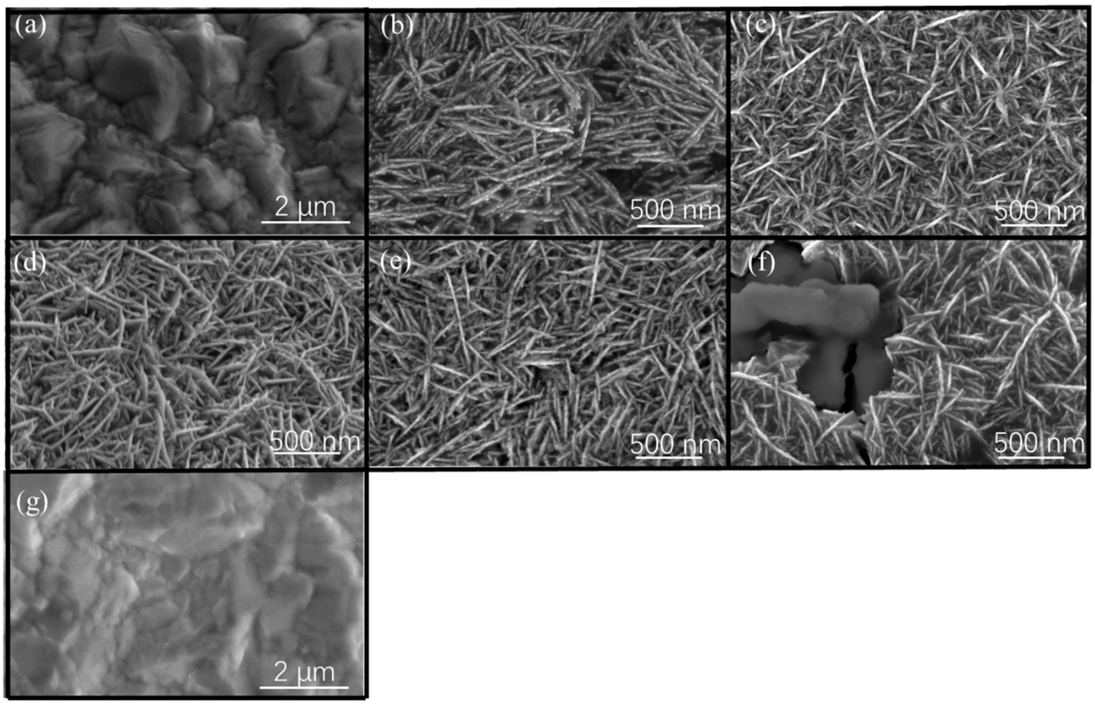


**Figure S1** SEM images of Ni@NiM prepared with different concentrations of $Ni(SO_3NH_2)_2 \cdot 4H_2O$; (a) 80 $g \cdot L^{-1}$; (b) 120 $g \cdot L^{-1}$; (c) 150 $g \cdot L^{-1}$; (d) 175 $g \cdot L^{-1}$; (e) 220 $g \cdot L^{-1}$; (f) 300 $g \cdot L^{-1}$; (g) 350 $g \cdot L^{-1}$.

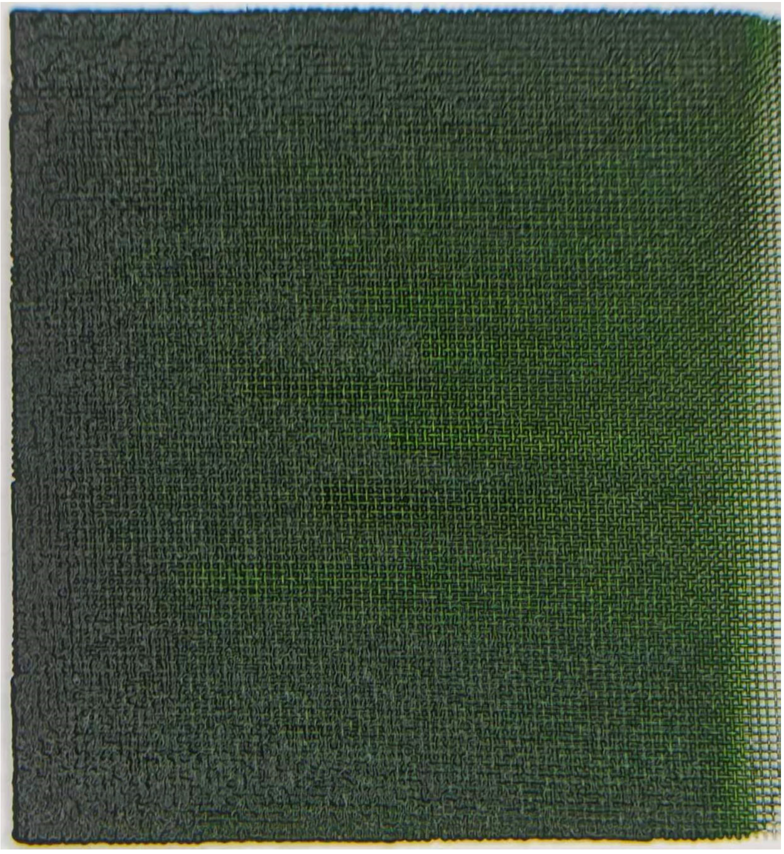

**Figure S2** SEM images of Ni@NiM prepared at 25 ℃

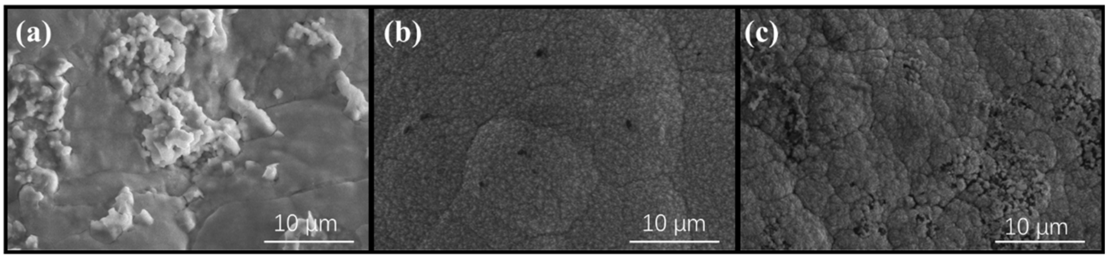


**Figure S3** SEM images of Ni@NiM prepared at different current densities; (a) 40 mA·cm$^{-2}$; (b) 80 mA·cm$^{-2}$; (c) 120 mA·cm$^{-2}$;

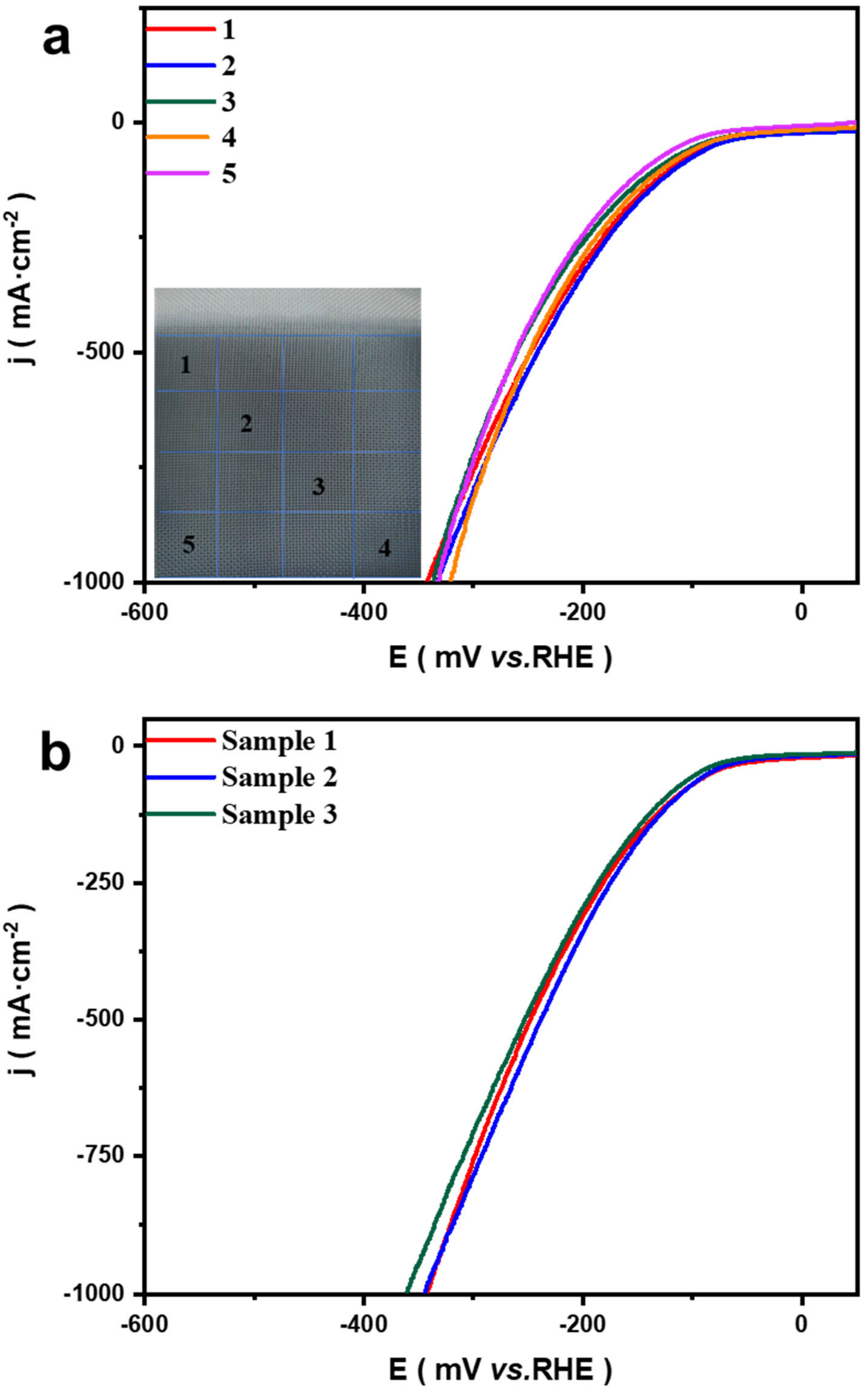


**Figure S4** (a) Uniformity test of Ni@NiM electrode; (b) Reproducibility test of Ni@NiM

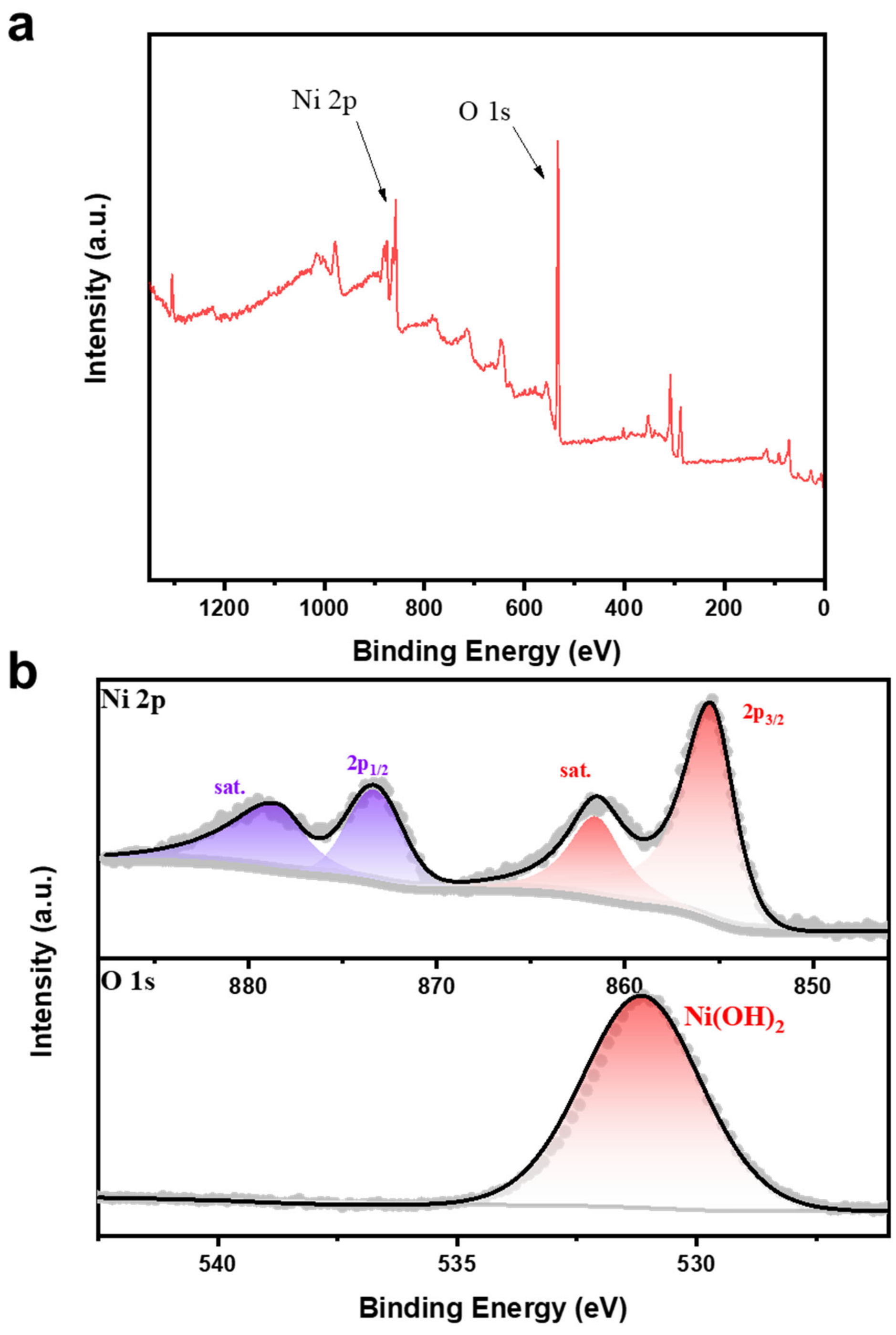


**Figure S5** (a) Survey XPS spectra of Ni@NiM after ALK water electrolysis; (b) High-resolution XPS spectra of Ni 2p and O 1s for Ni@NiM after ALK water electrolysis

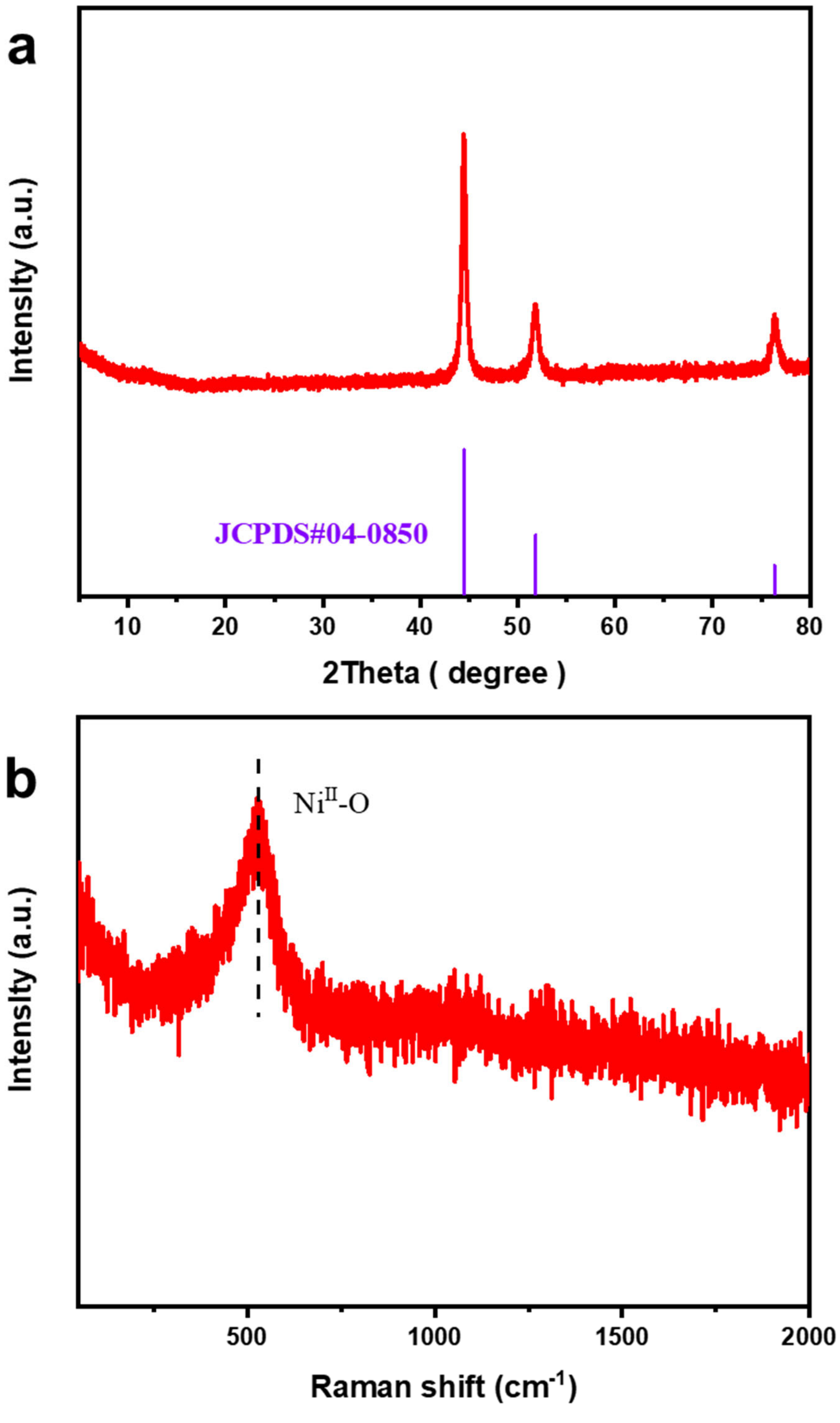


**Figure S6** (a) XRD patterns of Ni@NiM after ALK water electrolysis; (b) Raman spectra of Ni@NiM after ALK water electrolysis
